# Role and Impact of Cerebrolysin for Ischemic Stroke Care

**DOI:** 10.3390/jcm11051273

**Published:** 2022-02-25

**Authors:** Dafin F. Mureșanu, Livia Livinț Popa, Diana Chira, Victor Dăbală, Elian Hapca, Irina Vlad, Vitalie Văcăraș, Bogdan Ovidiu Popescu, Răzvan Cherecheș, Ștefan Strilciuc, Michael Brainin

**Affiliations:** 1 RoNeuro Institute for Neurological Research and Diagnostic, 400364 Cluj-Napoca, Romania; dafinm@ssnn.ro (D.F.M.); diana.chira@ssnn.ro (D.C.); victor.dabala95@gmail.com (V.D.); elianhapca@gmail.com (E.H.); irina.vlad001@gmail.com (I.V.); vvacaras@umfcluj.ro (V.V.); stefan.strilciuc@ssnn.ro (Ș.S.); 2 Department of Neuroscience, Iuliu Hatieganu University of Medicine and Pharmacy, 400083 Cluj-Napoca, Romania; 3 Department of Neuroscience, Carol Davila University of Medicine and Pharmacy, 050474 Bucharest, Romania; bogdan_ovidiu_popescu@yahoo.com; 4 Department of Public Health, Babes-Bolyai University, 400294 Cluj-Napoca, Romania; razvan.m.chereches@gmail.com; 5 Department of Clinical Neurosciences and Preventive Medicine, Danube University Krems, 3500 Krems, Austria; michael.brainin@donau-uni.ac.at

**Keywords:** stroke, Cerebrolysin, early motor rehabilitation, ischaemic stroke, neurorehabilitation, neuroprotection, neurotrophic activity, neuroplasticity, thrombolysis, recovery, cerebrovascular disorders, brain diseases, neuroprotective agents, protective agents

## Abstract

Stroke is still a significant health problem that affects millions of people worldwide, as it is the second-leading cause of death and the third-leading cause of disability. Many changes have occurred in the treatment of acute ischemic stroke. Although the innovative concepts of neuroprotection and neurorecovery have been vigorously investigated in a substantial number of clinical studies in the past, only a few trials managed to increase the number of promising outcomes with regard to the multidimensional construct of brain protection and rehabilitation. In terms of pharmacological therapies with proven benefits in the post-ischemic process, drugs with neurorestorative properties are thought to be effective in both the acute and chronic phases of stroke. One significant example is Cerebrolysin, a combination of amino acids and peptides that mimic the biological functions of neurotrophic factors, which has been shown to improve outcomes after ischemic stroke, while preserving a promising safety profile. The purpose of this paper is to offer an overview on the role and impact of Cerebrolysin for ischemic stroke care, by touching on various aspects, from its complex, multimodal and pleiotropic mechanism of action, to its efficacy and safety, as well as cost effectiveness.

## 1. Introduction

Stroke remains a serious health issue that impacts millions of individuals worldwide, representing the second-most common cause of mortality and the third-most common cause of disability [1]. Approximately 60–80% of all strokes are ischemic and result from thrombotic or embolic occlusion of a cerebral artery [2]. The management of acute ischemic stroke has undergone many changes. Regarding recanalization therapies such as thrombolysis and mechanical thrombectomy, the number of patients who may benefit from them is still low. Therefore, different therapeutic strategies have been developed, targeting the pathophysiological cascade that starts with ischemia and leads to irreversible tissue damage [3,4].

The innovative concepts of neuroprotection and neurorecovery have been actively researched in many clinical studies in the past. However, only a few trials in recent decades succeeded in increasing the number of positive results with reference to the broad concept of brain protection and rehabilitation, due to specific approaches that resulted in inconsistent evidence, therapeutic schemes that concentrated on suppressive strategies or the excessive research of the concept of monomodality (drugs that possess a single mechanism of action) [5].

Following an acute brain injury, there is an activation of an internal defensive array (known as the endogenous defense activity) consisting of two different pathways: neuroprotection (an immediate process, whose main target is the limitation of neuronal damage) and neurorecovery (which can be divided into three different branches—neurotrophicity, neuroplasticity and neurogenesis) [5]. 

The notion of multimodality, in neuropharmacological terms, refers to the binding of neuroprotection to the long-term reparatory processes that shape neuroregeneration, mirroring the physiological sequence of post-lesional endogenous regulation. Various attempts to integrate this concept were considered, with the use of monomodal drugs being ruled out as ineffective. Consequently, the multimodal agents with a pleiotropic neuroprotective effect in neurorehabilitation are considered a suitable solution based on current research [5].

Concerning the constellation of pharmacological therapies that could provide proven benefits in the post-ischemic process, the drugs with neurorestorative properties are effective in both the acute and chronic phases of stroke [6]. A significant advantage of neurorestorative drugs is their prolonged effectiveness, not being temporally restricted by pathologies such as brain ischemia. Because their availability exceeds the afferent therapeutic window of stroke (e.g., for tissue plasminogen activator-tPA), this type of pharmaceutical agent can be used for days, if not weeks, after an ischemic event. Restorative treatments should be coupled with rehabilitation, which likely acts synergistically to enhance neurological recovery [5]. Current data suggest that in over 70% of the stroke patients that benefit from thrombectomy and tPA, the perfusion rate of the affected tissues was still under the optimal parameters. Since complete tissue perfusion following such lesions is not feasible, many survivors develop further neurological deficits [6].

Cerebrolysin, a combination of amino acids and peptides that replicate biological effects of neurotrophic factors, is proven to exert beneficial outcomes when administered after ischemic stroke, while maintaining an encouraging safety profile. It reduces the number of procoagulant, prothrombotic and proinflammatory mediators, maintaining the normal function and health of the cerebral microvasculature after ischemia. An essential number of inflammatory cytokines is produced because of blood–brain barrier (BBB) injuries, caused not only by fibrin molecules, but also because of thrombolysis (with tPA). Therefore, the multimodal drug may enhance the therapeutic efficacy and safety of thrombolytic agents and thrombectomy, protecting the BBB [6].

As a result, future therapies may include neuroprotectants with more than one mechanism of action; therefore, multimodality should be systematically and intensively investigated, along with the discovery of novel agents and the thorough investigation of those that have demonstrated neuroprotective potential, not only before recanalization but also during the process. Subsequently, such actions could generate a further augmentation for the functional outcome, protecting the brain from both ischemia and reperfusion damage [6]. 

## 2. Concepts of Neuroprotection and Neuroregeneration

Stroke has immediate and long-term effects such as the impairment of movement, sensation, cognition, psychological and emotional functions, reducing independence and quality of life. Moreover, the neuroplastic changes that follow a cerebral infarction may occur over days, weeks, months or even years. However, despite all the progress in understanding the expansion of an ischemic event, the physiopathology of stroke is only partially known, the fundamental mechanisms of the brain that are related to its defense, protection and adaptation modulating processes are one of the enigmas and challenges of translational neurology [7].

Presently, the existence of an endogenous defense mechanism that incorporates neurobiological mechanisms, such as neuroprotection, neurotrophicity, neuroplasticity and neurogenesis, is already acknowledged. The central nervous system assesses controlling influence with modifying and repairing roles on different levels endogenously [8]. For this reason, their augmentation represents a therapeutic target [5].

**Figure 1:**
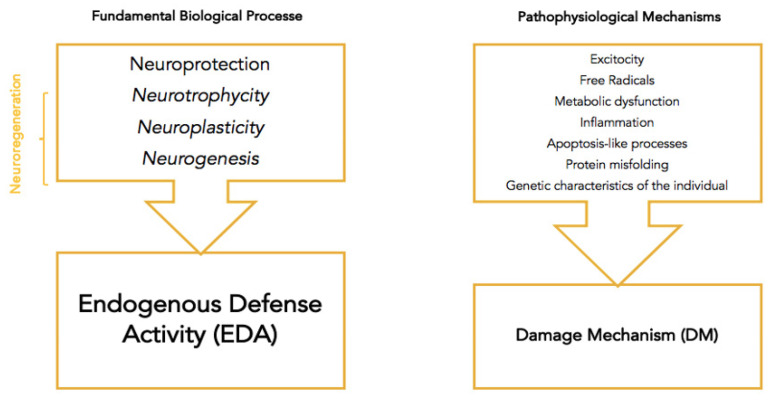
Endogenous defense activity (EDA) and damage mechanism (DM) [5].

The endogenous defense mechanism is activated following an acute injury such as an ischemic stroke through two anticorrelated mechanisms: the neuroprotection mechanism (with immediate action), and the (partially superposed) neuroregeneration mechanisms (neurotrophicity, neuroplasticity and neurogenesis) [9]. The two main anticorrelated processes aim in the first phase to reduce damage, leading to impairment, afterward aiming to repair the damage, followed by disability [9]. The fundamental biological processes and pathological mechanisms that comprise endogenous defense and damage mechanisms are summarized in Figure 1 [5]. 

Endogenous defense mechanisms consist of absolute and relative processes. Fundamental mechanisms lead to gene expression and protein synthesis, which play a restorative role. The other cell compounds, such as the cytoplasm, membrane or cytosol, are influenced by different (relative) mechanisms (e.g., ion channel blockers, agonists/antagonists of receptors, antioxidants, etc.), which determine neuroprotective activity expression [1,5,10].

Pharmacological neuroprotection follows a similar organization. Neurotrophic factors and neurotrophic-like molecules control the absolute mechanisms, while ionic channel blockers influence the relative mechanisms, agonists and antagonists of certain receptors, antioxidants and other monomodal therapeutic agents [1,5]. 

Neuroprotection, as part of the endogenous defense activity of the nervous system, is defined as the sum of all mechanisms allowing the neuron to functionally adapt against harmful factors. It is a short-term neurobiological process. The main aim of neurovascular protection is to preserve the components of the neurovascular unit, consisting of neurons, glial cells, endothelial cells, pericytes and matrix proteins. Any damage to parts of the neurovascular unit will lead to an apoptotic-like process, namely, anoikis [5,8,10,11]. 

The nervous system can change and adjust its activity, functions, and interrelations, in association with structural modifications, in response to the damaging action of different (intrinsic and extrinsic) factors, this capacity being termed neuroplasticity. Neuroplasticity plays an important role in the recovery process after stroke because of its reorganizational capacity, covering not only the neuronal structure, but also the functional aspects and their interrelation [1,10,12]. It overlaps with neurotrophicity and neuroprotection, sharing common mechanisms [10]. Not all physiopathological processes and the connections between these two processes are known. There is, however, a certain degree of overlap between them [13]. All of these fundamental biological processes, as mentioned before, have an absolute aspect and a relative aspect that substantially contrast [5,8]. 

Both excitotoxicity and inflammation possess a bivalent attribute: The role they have in developing an ischemic stroke is destructive and protective. While excitotoxicity occurs from the glutamate excess, and because of the consecutive surplus activation of the NMDA receptors, it leads, through proteolysis, to injury and cellular death, and in the case of a stroke, the inflammatory process expands through the activated immune cells. The two processes also share a common mechanism that is mediated through neurotrophic factors, which facilitate neuronal survival. Being part of neuroplasticity and neurotrophicity (for excitotoxicity), respectively (for inflammation), the endogenous actions of neuroprotection and neuroplasticity reveal the protective side of the whole activity (although certain conditions must be fulfilled, such as maintaining the proteolysis at an optimal level) [5,8,14,15,16,17,18,19].

Finally, of the pathophysiological mechanisms (unlike apoptosis, which represents a normal process in the human body, with the role of maintaining and controlling cellular populations), apoptotic-like processes always produce negative effects, this being the reason for the need of counteraction through exogen and endogen mechanisms [5,8,20,21,22]. In conclusion, there is a need to concentrate on an attempt to develop pharmacological therapies that act in ways that can discontinue the apoptotic-like processes and turn the balance of the excitotoxicity and inflammation towards their positive effects, as opposed to the negative effects [5,8,10,22].

The disability encountered after stroke varies greatly, from movement impairment to cognition and psycho-emotional status. Therefore, with the help of neurorehabilitation, there is an ongoing attempt related to amplifying the capacity of the neuroplasticity of the nervous system [11,22].

Neurogenesis represents the mechanism when new neurons are being produced from neural stem cells, and neurotrophicity describes the mechanism with which the cell constantly maintains its normal constitution and DNA expression [5,8,23].

Neurotrophic factors, neurotrophic-like factors and genetic parameters are all processes that initiate the endogenous defense activity, as well as its governance [5,10]. They act on the absolute mechanisms, that lead to gene expression and protein synthesis, which both play a restorative role. Other cell compounds, such as the cytoplasm, the membrane or the cytosol are influenced by different (relative) mechanisms (i.e., ion channel blockers, agonists/antagonists of receptors, antioxidants, etc.), which determine neuroprotective activity expression [5,10]. Consequently, the need to activate and accentuate the endogenous systems (both internal and pharmacological) resides in the efficient counter of the pathophysiological mechanisms. This exogenous augmentation can be achieved using various interventions, from pharmacological to psychological ones. The use of molecules that mirror the structure and function of endogenous molecules has been proven to be beneficial, considering the complex continuous processes in which the latter are involved. For instance, it is known that the first 72 h after an ischemic event are of utter importance, considering the relevance that they have in the mechanisms of neuroprotection and neurogenesis and neuroplasticity [5,10]. Neurorehabilitation strives to enhance these mechanisms for improved outcomes [5,7,8]. 

The physiopathological processes of stroke also occur in the stage between the vascular occlusion and cellular apoptosis, thus representing a target for pharmacological intervention, saving the neuronal population affected by the injury [10,11,24]. These defense mechanisms can be activated naturally or pharmacologically [10,11]. 

In recent years, many attempts have been made to develop neuroprotective drugs targeting the pathophysiological cascade that starts with ischemia and leads to irreversible tissue damage. Despite inconsistent results from numerous clinical trials, some of them have been successful (Citicoline, Cerebrolysin, Erythropoietin, etc.). The typical pattern that all these molecules share is that they exhibit a pleiotropic mechanism against the ischemic cascade [10,11,13]. 

**Figure 2:**
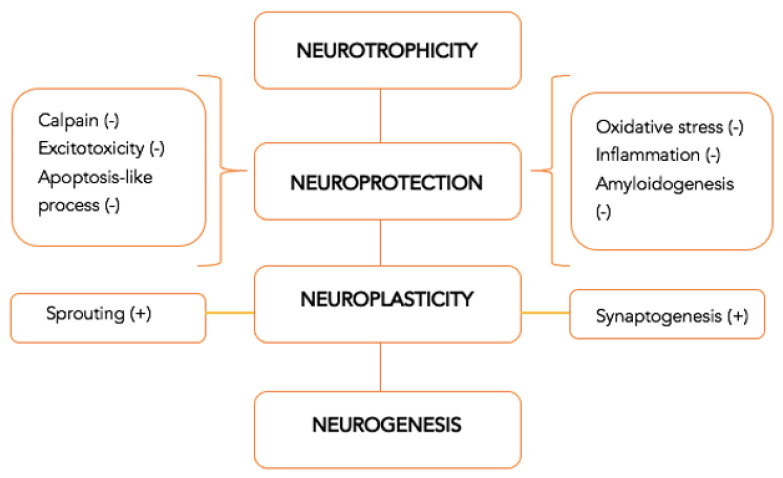
Multimodal drugs with pleiotropic neuroprotective effect—mechanism of action [5].

An important characteristic that an efficient neuroprotective drug should possess nowadays is multimodality, or the capacity to activate multiple biological mechanisms at the same time. The multimodal action is even more critical when the mechanisms of neuroplasticity and neurorecovery suffer from a long-term impairment, since many traditional pharmacological agents have an influence on a single mechanism [10]. The multimodal and pleiotropic effect provides the capacity to ensure, from a pharmacological perspective, the connection between the acute part, with a neuroprotection role, and the long-term part, with a regenerative role. The pharmacological consequences are summarized in Figure 2 [5].

The ensemble of pathophysiological mechanisms denotes the importance of the multimodal action of the pharmacological therapeutic agents. For instance, glutamate has both an excitotoxic action in the first minutes and hours post-stroke, as well as a neuroregenerative action after a few hours [5,13]. The control of this change, vital for the protection of the brain, is assigned to the action of multimodal pharmacological agents, triggering the shift from neuroprotection to neuroplasticity [5,13]. This is one of the reasons why timing the expression of the implicated genes with the pharmaceutical effect of this type of drug has an exponential role in the pathogenesis of ischemia [9]. 

At present, the general therapeutic term (unimodal pleiotropic) refers to the usage of drugs that work through increasing the action of EDA exogenously, focusing only on neuroprotection [5,9]. Therefore, there is a need to concentrate on the pharmacological performance and neuroprotective effects of the drugs with multimodal and pleiotropic activity, (i.e., the biological agents), as opposed to the unimodal acting ones, since the results that derived from the latter category are not consistent enough [5]. Based on this principle, Cerebrolysin is an example of an agent with a complex action mechanism. It possesses a proven multimodal, pleiotropic action, which provides not only immediate neuroprotection, but also long-term neuroregeneration, by activating endogenous responses that can be observed in a certain number of cerebrovascular and neurodegenerative diseases, including stroke [9]. 

Cerebrolysin is a complex compound, which is composed of peptides with active, neurotrophic activity and modulation, and free amino acids, which, through its multimodal action, promotes neurotrophic stimulation (through the survival and maintenance of the phenotype of highly differentiated cells), modulates neuroprotection against noxious agents (facilitating changes in the plasticity of neurons and synapses), and benefits the neuronal metabolism through an increase in resistance to hypoxic conditions and prevention of lactate accumulation (lactic acidosis) [9,25,26].

This multimodal action of Cerebrolysin was shown in animal and in vitro studies, consisting of a reduction in programmed cell death and free radicals’ development, the regulation of the inflammatory response, and a reduction in the toxic actions of neurotransmitters (excitotoxicity), all leading to neuroprotection; a reduction in regulating the increase in the number of synapses, leading to neuroplasticity; and a reduction in neurovascular reconstruction, leading to neurogenesis in the dentate gyrus [27,28,29,30,31,32,33].

This pharmaceutical agent increases neurogenesis and oligodendrogenesis, by activating the Sonic Hedgehog pathway, with a role in the evolution and structuring of the brain. One example is the Gli complex, which increases neurorecovery [34]. The capacity of Cerebrolysin to induce neurorecovery, along with the standard treatment of stroke, is the reason why this potent drug represents an efficient option as an add-on treatment for stroke rehabilitation [9]. It was suggested that Cerebrolysin has a more accentuated effect on neuroregeneration (neuroplasticity and neurorestoration) than on neuroprotection [13]. 

## 3. Clinical Trials

### 3.1. Efficacy of Cerebrolysin

Cerebrolysin has been the subject of multiple clinical trials, the majority of which have yielded encouraging results in terms of multimodal and pleiotropic activity. Various studies have shown that the intravenous administration of Cerebrolysin can improve the neurological outcomes of patients who have had an acute ischemic stroke, as well as its beneficial association with other pharmaceuticals and types of physical, occupational or speech therapies. Tran et al. studied the effect of Cerebrolysin with nootropics in the treatment of acute ischemic stroke (AIS) patients. Their results showed that Cerebrolysin, alone or in combination with other such pharmaceutical agents, was found to be safe and beneficial in the treatment of acute ischemia, in both the acute and recovery stages, indicating that it should be used in everyday clinical practice [35]. Chang et al. focused on the combination of Cerebrolysin with standardized rehabilitation therapy. The results showed that in individuals with severe motor impairment caused by acute ischemic stroke, conventional rehabilitation therapy combined with Cerebrolysin delivers extra benefits to conventional rehabilitation therapy alone in motor recovery [36]. An earlier study by Chang et al. (which used for the first-time neuroimaging for motor network plasticity evaluation when administering Cerebrolysin) revealed a positive influence of Cerebrolysin on cerebral tissue related to motor function, but no significant difference was found between the two groups [37]. Xue et al. conducted a clinical trial to test and assess the efficacy and safety of DL-3-n-butylphthalide (NBP) and Cerebrolysin in minimizing neurological and behavioral impairment after acute ischemic stroke. The findings of this study suggested that a 10-day treatment with NBP or Cerebrolysin could be used safely and may have favorable benefits in patients with AIS, especially in mild cases. However, NBP appeared to be better than Cerebrolysin at improving the short-term prognosis of acute ischemic stroke [38]. 

The study of Muresanu et al., CARS 1, showed a positive influence of Cerebrolysin on both functional and overall outcomes in early stroke rehabilitation. More precisely, participants who received the drug had better upper-extremity motor function at 90 days, than patients who received placebo [4]. Following CARS 1, a study of Guekht et al., CARS 2 aimed follow the design of the first trial, on a larger scale. However, this study did not support the findings of CARS 1, but Cerebrolysin was tolerated in its sample [39]. Razei et al. concentrated on just Cerebrolysin, aiming to see how it affected neurological results and the brain blood flow. The results showed that Cerebrolysin might help patients with acute focal ischemic stroke improve their neurological results, also affecting the pulsatility index (PI) of the middle cerebral artery [40]. Lang et al. conducted another trial to see if combining alteplase (rt-PA) with Cerebrolysin was safe and effective in reducing impairment following an acute ischemic stroke. They concluded that the neurotrophic agent combined with rt-PA was safe for the treatment of AIS, although it did not improve prognosis at 90 days. However, compared to the placebo group, considerably more patients had a favorable response in neurological outcome measures during the 10-day therapy period with Cerebrolysin [41]. Stan et al. assessed the efficiency of Cerebrolysin combined with post-stroke early rehabilitation, showing positive results in the cohort treated with Cerebrolysin. The authors reported improved overall neurological health and reduced impairment for the patients treated with Cerebrolysin. There were 28.5% more independent patients in the intervention group than the control group, showing that this study’s positive findings could be effectively used in contemporary clinical practice [42]. Heiss et al. aimed to see whether the treatment with Cerebrolysin was suitable and safe in patients with acute ischemic stroke. The validating endpoint in this trial revealed no differences between the treatment groups. However, a favorable outcome trend was observed in the heavily impacted patients treated with Cerebrolysin [43]. Additional information regarding the studies we have referred to may be found in Table 1. 

### 3.2. Safety Profile of Cerebrolysin

The safety of the neurotrophic drug Cerebrolysin was previously evaluated in various studies, presenting heterogeneous results regarding demographics, time of inclusion, administered dose and time of assessment/follow-up.

A trial published in 2012 reported a lower drop-out of patients from the study, due to side effects in the Cerebrolysin group, versus in the placebo group, concluding that there were no significant differences between the two categories. The comparison of the two clusters (Cerebrolysin and placebo) revealed no significant differences regarding side effects, severe side effects, and mortality. Additionally, no significant changes in vital parameters or laboratory test results were observed in patients treated with Cerebrolysin in the acute phase of ischemic stroke. It is noteworthy that during the analysis of the subcategory of patients with a NIHSS > 12 points, a lower death rate could be associated with the intervention group, compared to the placebo group [43].

Another randomized clinical trial that involved Cerebrolysin mentioned a low percentage (under 5%) of patients from the intervention group that were forced to leave the study due to adverse events. Of these, only two patients dropped out of the study prematurely due to adverse reactions. No deaths were reported in patients that received the neurotrophic treatment. Regarding adverse reactions, 2.9% of patients in this group and 6.7% of those receiving a placebo had a major side event with a complete resolution, considered to be unrelated to the administered agent. In the Cerebrolysin-treated group, severe side effects were mainly represented by acute myocardial infarction, severe peripheral ischemia, and renal colic, all of which underwent resolution during this study. According to the study protocol, the prolonged hospitalization of these patients, in addition to early rehabilitation, could be an explanation for the low rate of procedures regarding major secondary events. Additionally, relevant for this case was the absence of significant changes in the Cerebrolysin-treated group compared to the placebo-treated group, regarding vital and laboratory parameters [4].

A phase IV clinical trial, which included patients with moderate or severe motor dysfunction after a stroke, showed similar results between the intervention and placebo groups concerning the vital signs and laboratory values, while each group reported a single serious adverse event, the one recorded in the Cerebrolysin group being an episode of cholecystitis caused by gallstones, which remitted after some time [37].

Concerning the use of Cerebrolysin in patients who received intravenous thrombolytic treatment with Alteplase (rt-PA), no general increase in the frequency of deaths, severe side effects or adverse effects were reported, compared to the control group. In contrast to the other clinical trials, it is worth mentioning the differences in the timing of the administration of rt-PA, the shorter time between stroke-onset to rt-PA perfusion (onset to needle time), as well as the time of administration of Cerebrolysin between the placebo-group and the intervention group and the side effects (i.e., brain hemorrhage) that were attributed to the Alteplase. In this case, there were also no changes in vital or laboratory parameters related to the use of the neurotrophic drug [41]. 

A meta-analysis of nine randomized clinical trials reported a lower death rate in the Cerebrolysin group compared to the placebo group. When it comes to the calculated OR, it showed slightly lower results in the placebo group, but was statistically not significant. Moreover, the percentage of patients who reported severe side effects, and the one patient who reported at least one side effect, were comparable between the two groups. It is worth mentioning that only seven out of eight studies had available data regarding serious adverse events and adverse events [44]. 

Another meta-analysis focusing on the results of two corresponding stroke trials (CARS-1 and CARS-2—both presented in Table 1) assessed the tolerability and safety of Cerebrolysin on motor function recovery in patients after stroke. It was observed that the number of patients treated with Cerebrolysin that reported at least one adverse event (AE) was similar to the events of the patients in the placebo group. Moreover, most AEs were rated as mild in terms of severity. Regarding serious adverse events (SAEs), fewer than 5% of patients suffered from SAEs in each group and none of them was reported to be related to study medication [45]. In general, most of the adverse reactions reported in studies among patients who received Cerebrolysin were transient and could not be correlated with medication administration [13]. 

A 2021 systematic review and meta-analysis assessed the safety of administrating Cerebrolysin after acute ischemic stroke compared to a placebo. Based on the analysis of 12 randomized controlled trials, the safety profile of Cerebrolysin was assessed using not only the 12 RCT study population (2202 patients) but also subgroups. Considering the SAEs, death, AEs, and NF-SAEs of Cerebrolysin compared to a placebo, Cerebrolysin showed a good safety profile. Moreover, a trend toward SAE reduction was observed in the Cerebrolysin subgroup of patients with moderate-to-severe stroke, compared to the placebo [46].

Lastly, another systematic review assessed the benefits of Cerebrolysin in the treatment of acute ischemic stroke and the potential risks that the agent could possess during therapy. The results of the review concluded that Cerebrolysin made little to no difference regarding the risk of death from any cause after an ischemic stroke, the total number of subjects with SAE, the number of SAEs that resulted in death and the total number of patients who had any less SAEs. Referring to the combination of Cerebrolysin and standard therapy, the review reported an increase in the number of patients who had SAEs that did not result in death, compared to the groups who were assigned to standard therapy (alone or with placebo) (4 studies—1435 patients). Finally, the review did not find sufficient evidence regarding the risk of mortality and the need of continuous care at the end of the admitted studies [47].

All things considered, the safety of Cerebrolysin was studied in several different trials. There were no major differences registered compared to the placebo in terms of adverse effects (serious or fatal). Furthermore, there were no significant changes in vital and laboratory parameters. 

## 4. Observational Research—Effectiveness Studies

Often, an intervention is analyzed in terms of efficacy and effectiveness by clinicians and policymakers. One of the differences between these concepts is that efficacy trials (explanatory trials) play a role in determining whether the intervention produces the expected result, under ideal conditions. On the other hand, effectiveness trials (pragmatic trials) have the role of quantifying the degree of the beneficial effect in the context of “real world” circumstances. As a result, it is important to acknowledge that study designs and effectiveness trial hypotheses are developed by taking into consideration routine clinical practice and outcomes from interest in clinical decisions [48]. An extensive literature research, using Pubmed, was conducted with a focus on observational studies of the effectiveness of Cerebrolysin in stroke. Very few observational studies were identified; the most significant ones are presented below.

A retrospective study on the effectiveness of Cerebrolysin in post-stroke spasticity, was conducted on 50 patients (23 in the Cerebrolysin group and 27 in the control group). They received Cerebrolysin for 30 days, with 10 mL/day administered intramuscularly. Additionally, both groups participated for a minimum of 2 times a week in standardized physical and occupational rehabilitation therapy. Efficacy at day 30 was assessed with modified Ashworth scale (MAS), manual muscle testing (MMT) and modified Rankin scale. Cerebrolysin proved safe and effective; it showed a significant improvement in limb spasticity compared to the control group. In the Cerebrolysin group, strength and global function were also improved [49]. 

In a study by Kim et al., the effectiveness of Cerebrolysin on the state of consciousness was assessed in stroke patients with a minimally conscious state (MCS) in an observational retrospective study. Seventy-five patients with ischemic and/or hemorrhagic stroke with MCS according to the Coma Recovery Scale-Revised (CRS-R) were included during a period of 3 years. The Cerebrolysin group consisted of patients that had received Cerebrolysin 10 mL iv for a minimum of 20 days; the control group were patients with no Cerebrolysin administration. In addition, they received rehabilitation—physical and occupational therapy. Patients were assessed at discharge (~2 months) according to the Coma Recovery Scale-Revised (CRS-R) and, after eliminating confounders, showed a significant improvement, especially in the Oromotor and Arousal subscales. Furthermore, no safety issues were identified [50]. Based on our queries, the most significant studies identified in the literature research were interventional.

## 5. Cost-Effectiveness Research

Despite its high physical and cognitive burden, stroke also causes a tremendous economic burden worldwide. Stroke accounts for around 3 to 4 percent of the overall healthcare costs in Western countries [51]. Based on a recent systematic review by Rochman et al., it was observed that direct medical costs accounted for 86.2 percent and 13.8 percent of the overall cost [52]. These costs include medication, which plays an enormous part in treating stroke. 

Cost-effectiveness analyses (CEAs) are reliable tools for health system stakeholders (both policy and decision makers) assessing the value (characterized by costs and outcomes within a predefined time-point) of pharmacological therapies [53,54]. CEA is typically accompanied by a budget impact analysis (BIA), which evaluates how affordable a pharmacological intervention is in a system with limited resources, before approval or reimbursement processes. Given the high competition for limited financial means within each country and the particularities of national insurance systems, CEAs and BIAs are recommended to be conducted for each alternative intervention [54]. Few economic evaluations have assessed how cost-effective the pharmacological therapy with Cerebrolysin is or its impact on national health budgets.

Kulikov and Abdrashitova et al. aimed to evaluate the cost effectiveness of Cerebrolysin against standard therapy for patients diagnosed with moderate or severe stroke. Based on the reported results, Cerebrolysin was the dominant therapy, as cost-effectiveness ratios (CER) were lower (EUR 6920) than standard therapy (EUR 9287) [55]. Kulikov and Abdrashitova et al. have also been developing a complementary budget impact analysis (BIA) since 2015, which showed that Cerebrolysin has lower direct costs associated with medical care (inpatient and outpatient care, pharmacotherapy, emergency care and neurorehabilitation) and indirect costs (loss of productivity caused by sick leave, disability, and death). The costs incurred by stroke patients included in the Cerebrolysin group were reduced by EUR 1314 compared to the cost incurred by patients receiving standard therapy (EUR 7552) [56]. Walter et al. showed that, in patients diagnosed with acute ischemic hemispheric stroke and treated with a combination of alteplase and Cerebrolysin, lower costs were reported (EUR 61,468.67) compared with patients receiving only alteplase. The treatment strategy involving neuroprotective agents had reduced costs attributable to acute ischemic CVA care and nursing homes [57]. 

While existing studies are scarce, there is a signal that Cerebrolysin is probably cost-effective, with the potential to reduce the economic burden on national budgets, both as standard treatments in patients with different levels of severity [55,56] or in combination with another pharmacological therapy (i.e., alteplase) [57] in countries with different types of health insurance [58,59]. 

## 6. Cerebrolysin Recommendations in Guidelines

Cerebrolysin has been featured in different guidelines regarding its administration in the acute phase of stroke, as well as during the chronic phase, for its evidence-based role in rehabilitation [46]. In 2020, Cerebrolysin was recommended in the Stroke Rehabilitation Clinician Handbook, to be administered in the rehabilitation process of the hemiplegic upper limb, stating the possibility of improvement of motor function, agility, and self-reliance during daily activities using Cerebrolysin. The recommendation was based on randomized controlled trials, with Cerebrolysin being administered in 30 mL dosage on 70 mL saline, once daily, intravenously for three weeks combined with physical or occupational therapy, or the same dosage and administration route as before, but for six weeks combined with standard rehabilitative therapy [60]. 

Cerebrolysin was also recommended as part of the pharmacological rehabilitation of the motor-deficient upper limb after stroke in the 2020 revised guideline of the German Society of Neurorehabilitation regarding the rehabilitation of the paretic upper limb. The recommendation was evidence- and consensus-based, with the group in charge of the guideline’s conceptualization, The German Society for Neurorehabilitation. The guideline recommends the administration of Cerebrolysin in the acute and subacute phase of stroke in patients with a pertinent upper limb motor deficit, ideally 24 to 72 h after stroke, intravenously, daily, for 21 days (if tolerated). The aim of using Cerebrolysin is to enhance upper limb motor capacity and overall functional improvement, together with rehabilitation. Currently, Cerebrolysin is approved in Austria [61]. 

The 2021 European Academy of Neurology and European Federation of Neurorehabilitation Societies guideline on pharmacological support in early motor rehabilitation after acute ischaemic stroke recommends using two pharmacological agents, namely, Cerebrolysin and Citalopram. Cerebrolysin is recommended as a pharmacological add-on to the early motor rehabilitation in acute stroke with a dose of 30 mL/day administered intravenously, for a minimum of 10 days [62]. The existing evidence regarding the role and efficacy of Cerebrolysin in acute stroke was assessed and recommendations were made, using the Grading of Recommendations Assessment, Development and Evaluation (GRADE) framework, by the task force comprised by representatives from both the European Academy of Neurology (EAN) and the European Federation of Neurorehabilitation (EFNR) and from six European countries [62]. Six meta-analytics pathways (PICOs) were applied for scale classification: with the primary and secondary being the early motor performance at 30 days and at 90 days. Neurological function at 30 and 90 days and global functional outcome at 30 and 90 days, respectively, were considered for the safety profile when making the recommendations. Data extraction was followed by a meta-analysis [62].

In the AHA/ASA 2019 Guidelines for the Early Management of Patients with Acute Ischemic Stroke (2019 Update to the 2018 Guideline), aspects of neuroprotective therapy are briefly stated in the “General Supportive Care and Emergency Treatment” section. Although an A-Level of Evidence (LOE) was associated with the administration of neuroprotective agents, no beneficial effect regarding the patient’s status was considered, therefore falling in Class of Recommendation (COR) III. Therefore, the guideline does not recommend the administration of any medicative and non-medicative agents with an assumed neuroprotective activity in the acute phase of ischemic stroke. Nevertheless, no randomized controlled trial (RCT) of Cerebrolysin was specifically included in the assessment process, which encompassed high-dose albumin and magnesium infusion only [63]. A similar approach to recommendation for post-stroke pharmacological intervention to aid neurorehabilitation is present in the 2016 AHA/ASA guidelines on Stroke Rehabilitation and Recovery [64].

## 7. Conclusions

Given the high frequency and poor prognosis of acute ischemic stroke, it is vital to discover effective medicines to help people with the disease enhance their neurological and cognitive skills. Based on the complex pathophysiological cascade associated with brain ischemia, a multimodal approach, targeting various critical mechanisms, appears to be a key future approach to enhance therapy. Agents that exert an endogenous activity should be considered the essential elements for the effective development of therapies that target the protection and recovery of the brain from an acute CNS lesion. These agents have the potential to act at the DNA level, with the activation of synergic molecular mechanisms that provide the reintegration of homeostasis of the damaged neurological tissues [5]. The multimodal and pleiotropic action of Cerebrolysin leads to immediate neuroprotection and long-term regeneration [9]. An essential advantage of this neuroprotective drug is the argument that it can be widely used without relevant restrictions. Furthermore, there is no time window limitation for the drug administration, showing a high safety profile and being well-tolerated. Equally important, there is a considerable body of evidence that Cerebrolysin protects the brain against the impact of the ischemic cascade and supports the whole neuronal reorganization process [10,13].

Brain plasticity is the most important process of a synaptic phenomenon that is mainly a stimulus-dependent process. Recovery after stroke is a time-consuming and complex process, in which specific large brain lesions require not only new anatomical substrates, but also the renewing or creation of new network connections. It is important to understand that stroke is not only a regional impairment of the infarcted area, but its involvement in the distribution of whole-brain networks results in a broad spectrum of dysfunction and disability [5]. At the same time, for the functional outcome after stroke, acute rehabilitation, emphasizing timing and intensity, is of utmost importance. While early mobilization after stroke is recommended in clinical guidelines, it remains controversial, since it can also cause harm when the intensity is too high [64].

Neurological disorders represent a major burden globally, with regional and worldwide variability. Unfortunately, regardless of region, stroke predominates, especially when taking DALY (disability-adjusted life year) into account. The aim of the medical intervention in stroke, ranging from primary prevention to acute treatment, hospitalization, secondary prevention, and rehabilitation, is to lessen stroke occurrence, disability, and mortality [62]. The crucial role of proper early motor rehabilitation to reduce the debilitating consequences of stroke could be assisted by pharmacological support [13,60,61,62]. Further work is required, especially on understanding the balance between excitatory and inhibitory signals, determining the effects that injury has on them and, of course, identifying those targets that can efficiently control and maintain the equilibrium. Such a level of information would facilitate an accurate modulation of the neuronal network, leading to the activation of a localized mechanism of plasticity, while preserving stability in other areas [65].

Cerebrolysin is already contributing to the pharmacological armamentarium that clinicians have at their disposal in many countries worldwide [9,10,13]. Despite the prolific literature that has been published on the efficacy and safety of the add-on intervention for post-stroke neurorehabilitation, some limitations of clinical studies (e.g., small sample sizes) warrant additional investigation via large, high-quality confirmatory trials, as well as clinical and cost-effectiveness studies [4,35,36,37,38,39,40,41,42,43]. Improving this body of evidence in the future will impact current clinical guideline recommendations for Cerebrolysin after ischemic stroke, likely clarifying divergent opinions on this topic [60,61,62].

## Figures and Tables

**Table 1:** Trials involving the effect of Cerebrolysin on acute ischemic stroke in the past 9 years.

Article	Intervention	Case Numbers	Type	Methods	Primary Endpoint	Secondary Endpoint	Results
Tran et al., 2021	Cerebrolysin + Nootropics	190-Cerebrolysin 86-Placebo	Non-interventional, controlled, open-label, prospective and multicenter study	Cerebrolysin (10 mL), other nootropics, or a combination of both	Modified Rankin Scale (mRS)	National Institutes of Health Stroke Scale (NIHSS), Montreal Cognitive Assessment (MoCA)	mRS: improvement in Cerebrolysin 81.6%, combination 93.4%/placebo 43% NIHSS: good responders Cerebrolysin 77.5%, combination 92.5%/placebo 47.6% MOCA: scores Cerebrolysin 23.3 ± 4.8, combination: 23.7 ± 4.1/placebo 15.9 ± 7.7
Chang et al., 2021	Cerebrolysin + Standardized rehabilitation therapy	59-Cerebrolysin 51-Placebo	Combined data from the both phase IV prospective, multicenter, randomized, double-blind, placebo-controlled trials	Cerebrolysin or placebo with standardized rehabilitation therapy for 21-day treatment course	Fugl–Meyer Assessment	Motor Evoked Potential (MEP)	FMA-upper limb: T1–T2 significant improvement in Cerebrolysingroup MEP T1: positive response Cerebrolysin 33.9%/placebo 27.5% MEP T2: increased both groups, Cerebrolysin 42.4%/placebo 35.3%
Stan et al., 2017	Cerebrolysin	30-Cerebrolysin 30-Placebo	Prospective, randomized, double-blind, placebo-controlled	30 mL/day Cerebrolysin or to placebo for 10 consecutive days, starting in the first 24–48 h after stroke	National Institutes of Health Stroke Scale (NIHSS)	Modified Rankin Scale (mRS)	NIHSS higher scores in the Cerebrolysin group day 10: MW = 0.79 day 30: MW = 0.75 mRS day 30: independent patients in Cerebrolysin group: 73.33%/placebo: 44.83%
Chang et al., 2016	Cerebrolysin	35-Cerebrolysin 35-Placebo	Prospective, multicenter, randomized, double-blind, placebo-controlled, parallel-group study	30 mL/day Cerebrolysin or to placebo for 21 days	Fugl–Meyer Assessment	National Institutes of Health Stroke Scale (NIHSS)	no significant difference was found between the two groupsTotal FMA: 42 Cerebrolysin, 42.2 placebo NIHSS: 8.4 Cerebrolysin, 7 placebos
Xue et al., 2016	Cerebrolysin vs. DL-3-n-butylphthalide (NBP)	20-Cerebrolysin 20-Placebo 20-NBP	Randomized, double-blind trial	10-day intravenous administration of NBP (Cerebrolysin or placebo)	National Institutes of Health Stroke Scale (NIHSS) and Barthel Index (BI)	-	NIHSS day 21: lower scores for Cerebrolysin and NBP group BI day 21: higher scores for Cerebrolysin and NBP group
Muresanu et al., 2015	Cerebrolysin+ Standardized rehabilitation program	104-Cerebrolysin 104-Placebo	Prospective, randomized, double-blind, placebo-controlled, multicenter, parallel-group study	Cerebrolysin (30 mL/d) or a placebo (saline) once daily for 21 days, beginning at 24 to 72 h after stroke onset + Standardized rehabilitation program for 21 days	Action Research Arm Test	Modified Rankin Scale (mRS)	ARAT day 90: an increase in 92.3% of patients in Cerebrolysin group/84.2% placebo mRS: score of. 0–1 in 42.3% patients in Cerebrolysin group/14.9% placebo
Guekht et al., 2015	Cerebrolysin	120-Cerebrolysin 120-Placebo	Prospective, randomized, double-blind, placebo-controlled, multicenter, parallel-group study	Cerebrolysin (30 mL/d) or a placebo (saline)	Action Research Arm Test	Gait velocity, fine motor function, global neurological status, disability, quality of life, neglect	No end points showed significant improvement at 90 days for Cerebrolysin group mild baseline levels of impairment showed improvement after 90 days in placebo group
Razei et al., 2014	Cerebrolysin	23-Cerebrolysin 23-Placebo	Randomized, double-blinded, placebo-controlled trial	Cerebrolysin (30 mL) diluted in normal saline daily or Normal saline alone, adjunct to 100 mg of aspirin daily for 10 days	National Institutes of Health Stroke Scale (NIHSS)	Mean flow velocity and PI of cerebral arteries	NIHSS day 60 and 90: lower values in the Cerebrolysin group mean flow velocity day 30: higher in the placebo group median = 53, Cerebrolysin median = 45PI lower in the Cerebrolysin group 0.85/placebo 1.1
Lang et al. 2013	Cerebrolysin + Alteplase	60-Cerebrolysin 59-Placebo	Placebo-controlled, double-blind trial	Cerebrolysin (30 mL) or placebo (1 h after thrombolytic treatment) starting within three-hours after onset of symptoms, given for 10 consecutive days	Modified Rankin Scale (mRS)	National Institutes of Health Stroke Scale (NIHSS), Glasgow Outcome Scale (GOS), Barthel Index (BI)	mRS day 90: no significant improvement in Cerebrolysin group vs. placebo NIHSS, GOS, BI: no significant improvement in Cerebrolysin group vs. placebo
Heiss et al., 2012	Cerebrolysin	529-Cerebrolysin 541-Placebo	Double-blind, placebo-controlled randomized clinical trial	30 mL Cerebrolysin daily or placebo (saline solution) given as intravenous infusion for 10 days in addition to aspirin (100 mg daily)	Modified Rankin Scale (mRS), National Institutes of Health Stroke Scale (NIHSS), Barthel Index (BI)	Responder analysis and global test	NIHSS day 90: improved by 6 Cerebrolysin/5 placebo BI: 30 for both groups mRS: 2 for both groups global test MW = 0.50 CI = 0.46

## Data Availability

Not applicable.
